# Establishment of the First Institution-Based Poison Information Center in Nepal Through a Multilateral International Partnership

**DOI:** 10.9745/GHSP-D-24-00142

**Published:** 2024-10-29

**Authors:** Ramu Kharel, Rakesh Ghimire, Rajesh Sharma, Kabin Maleku, Adam R. Aluisio, Ziad Kazzi

**Affiliations:** a Department of Emergency Medicine, Warren Alpert School of Brown University, Providence, RI, USA.; b Department of Clinical Pharmacology, Tribhuvan University Teaching Hospital, Kathmandu, Nepal.; c Drug and Toxicology Center, Poison Information Center, Tribhuvan University Teaching Hospital, Kathmandu, Nepal.; d ASK Foundation, Kathmandu, Nepal.; e Department of Emergency Medicine, Emory University School of Medicine, Atlanta, GA, USA.

## Abstract

Toxicological emergencies present a significant health challenge in Nepal. Despite the high burden, the country has inadequate formal toxicology training, medical toxicology expertise, and adequate poison control infrastructure. In recognition of this need, the Nepal Poison Information Center (PIC) was established as a collaborative effort involving local and international partners. Through a comprehensive partnership framework, the Nepal PIC provides 24 hours a day, 7 days a week expert guidance to health care workers, conducts educational webinars, and engages in research. Initial data from the pilot phase indicate successful consultation delivery. Challenges include bureaucratic hurdles and the need for sustainable funding. Despite these challenges, the Nepal PIC demonstrates early feasibility and potential for expansion into a comprehensive toxicology center, contributing to the advancement of clinical toxicology in Nepal. Long-term sustainability relies on governmental support and continued advocacy efforts.

## Abstract (precis)

Through an innovative local and international partnership model, the first institution-based poison information center in Nepal was established in response to a mandate from the Nepal government, the high burden of toxicological emergencies in Nepal, and the known economic and health benefits of poison information centers.

## Key Messages

- Nepal has a high burden of toxicological emergencies but has grossly inadequate expertise and infrastructure for toxicology care.
- Nepal’s first institution-based poison information center (Nepal-PIC) was established through a local and international partnership with a focus on service delivery, education, and research.
- Early data show the utility and benefit of the Nepal PIC, but the sustainability of the Nepal PIC will ultimately depend on the local institution and government buy-in and funding.

## INTRODUCTION

Toxicological emergencies represent a significant health burden in Nepal, with self-harm through pesticides contributing to the largest proportion.^1^ According to the Nepal Burden of Disease 2019 report, 7.8% of all deaths were attributed to injuries. Self-harm, including ingestion of analgesics, antipyretics, antirheumatics, gases, vapors, hanging, strangulation, and suffocation, accounted for 1.8% of all deaths in 2019.^2^ Among poisonings, organophosphate pesticides are the most common cause, alongside other highly hazardous pesticides.^3^ The use of pesticides has specifically risen in suicide, with a 13-fold increase in suicide by pesticide ingestion between 1980 and 2018, accounting for nearly 23% of all suicides during this period.^4^ A retrospective review of medical records at 10 hospitals and 2 forensic laboratories in Nepal found that 95.8% of all pesticide poisonings were due to self-poisoning.^5^ Other major causes of poisoning in Nepal include drugs, such as paracetamol and its combinations, benzodiazepines, salicylates, amitriptyline, and opioids.^6^^,^^7^ Snakebite envenomation is another significant cause of toxicological morbidity and mortality in Nepal, affecting all age groups.^8^ Eighty-nine species of snakes have been recorded in Nepal, of which 17 species are venomous.^9^

Toxicology care and services in Nepal, as in most low and middle-income countries, are limited. There is no formal training in medical toxicology available to date,^10^ and the only poison information center (PIC), the Nepal Drug and Poison Information Center, is a noninstitutional, nongovernmental, privately run hotline with support from the Ohio Poison Center. Current data on its service scope and availability was not available.^11^ An observational study found that Emergency and Forensic Medicine and Toxicology residents and nurses in Nepal had insufficient exposure to toxicology education and lacked the skills to manage these cases.^12^ Care for poisoned patients is primarily provided based on the experience of health care providers with managing toxicology cases; there are currently no licensed medical toxicology experts working in Nepal. Inadequate availability of formal training, unavailability of information on care and patient management, and limited availability of antitoxins and antivenoms are some of the identified challenges and barriers to toxicological care in Nepal.^13^

Inadequate availability of formal training, unavailability of information on care and patient management, and limited availability of antitoxins and antivenoms are some of the identified challenges and barriers to toxicological care in Nepal.

Recognizing the need for a national poison center, the Government of Nepal recommended the development of a strategy for a national poison center as one of the top priorities to strengthen Nepal’s emergency care.^14^ Lack of trained human resources and poor coordination in establishing poison control programs were cited as major barriers to the development of sustainable poison control. Furthermore, prior cost-effectiveness analyses from various countries have shown that poison centers are economically viable as they lower health care expenditures.^15^ With a national government mandate, a clear need to have nationalized and standard resources in the care of toxicological emergencies, and established health and economic benefits, Nepal’s first institutional PIC (Nepal PIC) was established at the largest government teaching hospital in Nepal. We describe the process of the establishment, current progress, challenges, and outlook.

## THE POISON INFORMATION CENTER DESIGN: PARTNERSHIP, LOGISTICS, AND WORK PROGRESS

An emergency care and public health expert from the United States with deep-rooted connections and research experience in Nepal conceptualized the idea and served as the principal investigator (PI) for the Nepal PIC model. As part of the COVID-19 pandemic response, the PI collaborated with a local Nepali nonprofit, ASK Foundation, to establish a hotline supporting COVID-19 patients across the country.^16^^,^^17^ This telehealth infrastructure and experience drove the idea for the Nepal PIC. Several key factors were considered in establishing a PIC in Nepal: (1) a dearth of medical toxicology expertise in Nepal; (2) the absence of standard national toxicology care protocols; (3) limited accessible data on toxicology morbidity and mortality and a lack of a centralized database of available antidotes, antivenoms, or other essential medications; and (4) the feasibility of a PIC in Nepal had not been previously studied.

Taking these considerations into account, the Nepal PIC design was developed through consultations with various medical toxicology experts in the United States, as well as clinicians, pharmacologists, and government bodies in Nepal. To first study the feasibility of an institutional PIC in Nepal, a pilot study was designed.

### Ethical Approval

Ethical approval was obtained from the Lifespan Institutional Review Board and the Nepal Health Research Council.

### Partnership Model

**FIGURE 1:**
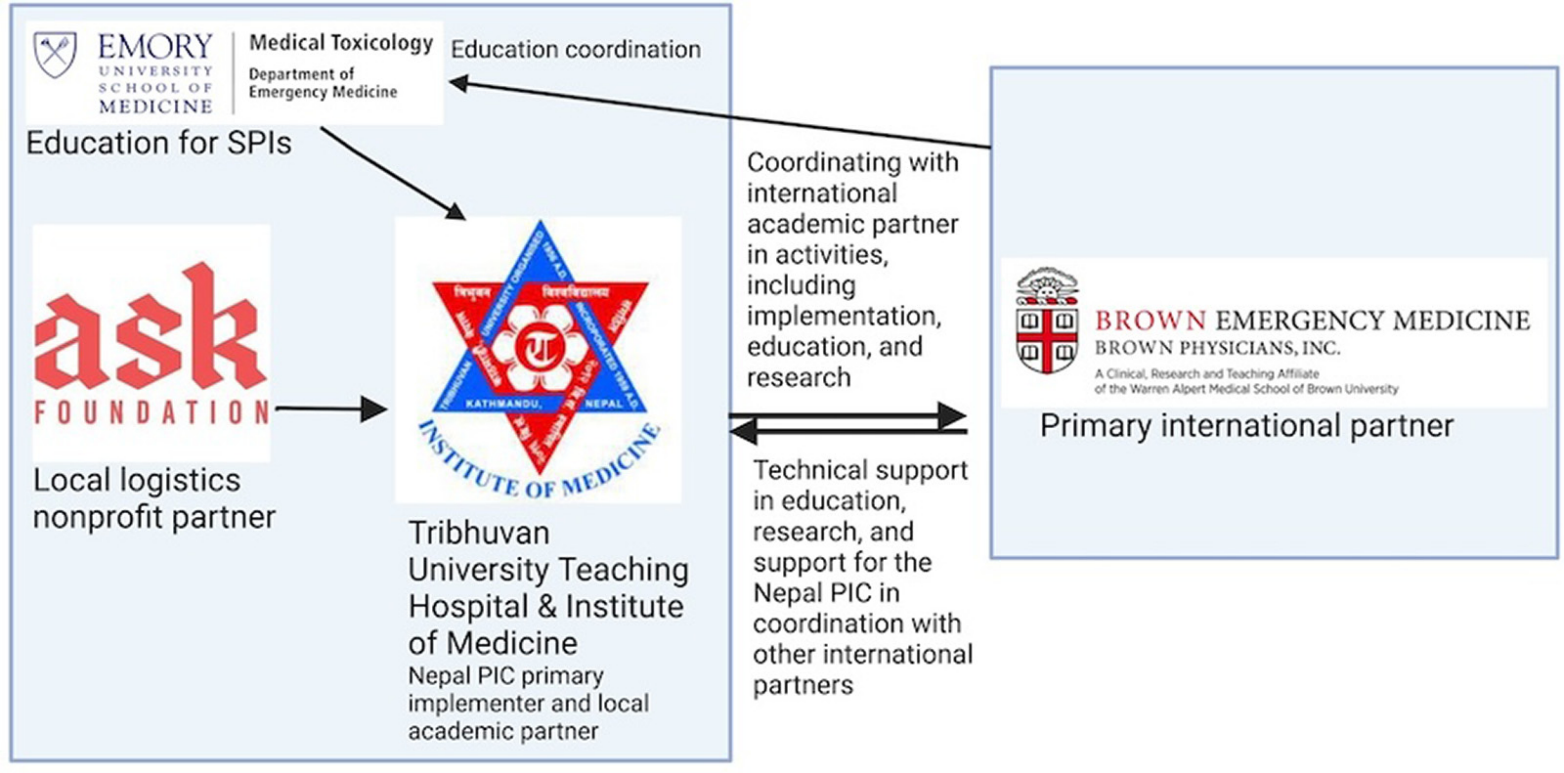
Nepal PIC Pilot Partnership Framework^a^ Abbreviations: PIC, poison information center; SPI, specialist in poison information. ^a^Brown Emergency Medicine serves as lead international coordinator. Tribhuvan University Teaching Hospital/Institute of Medicine and ASK Foundation serve as primary in-country partners.

Figure 1 illustrates the comprehensive partnership framework for the Nepal PIC. The PI led the pilot study, overseeing overall coordination and partner finalization and providing overall scientific and operational direction. After careful consideration of multiple potential partners in Nepal, a 3-way memorandum of understanding was signed on April 26, 2023, between the PI; Tribhuvan University Teaching Hospital (TUTH), Nepal’s largest academic hospital, which served as the local implementing and academic partner; and ASK Foundation, which had experience in telehealth and prior experience with hotline-based care, serving as the logistical support partner in the establishment of the PIC. The Clinical Pharmacology Department at TUTH joined as the primary implementation partner. This department had previously housed the Drug Information Unit at TUTH and has faculty who had conducted significant research on pesticides in Nepal.^5^^,^^17^ ASK Foundation’s role included operational and equipment support, dissemination, and fundraising. Additionally, Emory University’s Department of Emergency Medicine Medical Toxicology section joined as the education partner to help train and certify the specialists in poison information (SPIs) who would be staffing the PIC.

### Nepal Poison Information Center Activities

**FIGURE 2:**
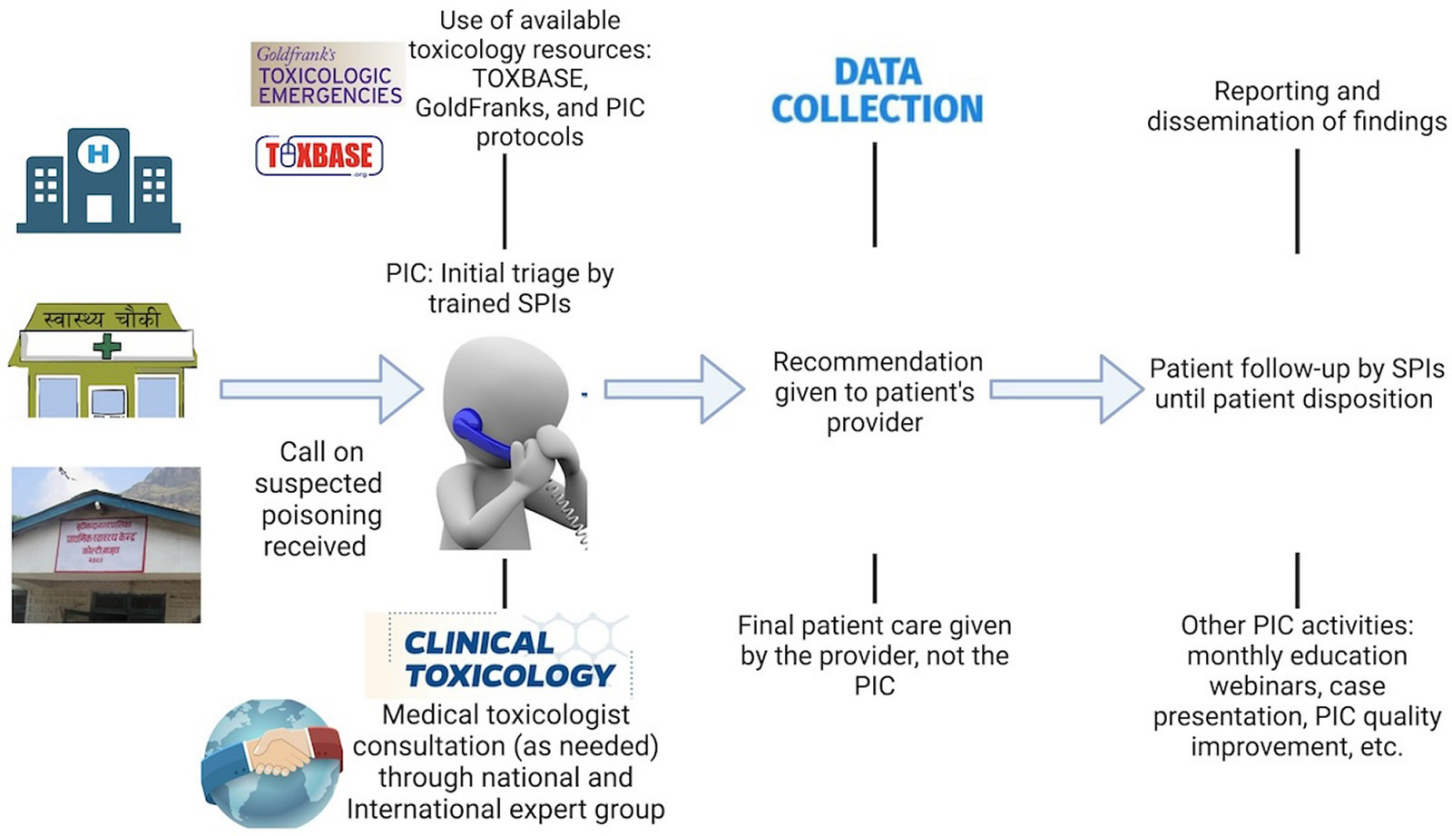
Nepal PIC Workflow Abbreviations: PIC, poison information center; SPI, specialist in poison information.

Figure 2 provides an overview of the workflow of the Nepal PIC. During the pilot phase, which began in December 2023 and is ongoing, the Nepal PIC is exclusively accessible to health care workers (HCWs) across Nepal. Any HCW in Nepal who has a patient with a suspected or confirmed case of toxicological exposure can contact the Nepal PIC using a locally accessible phone number. The PIC is staffed by trained SPIs who are available to answer calls 24 hours a day, 7 days a week. The SPIs are medical graduates (MBBS) who have undergone training provided by the educational partner and have undergone weekly training through various international and local opportunities. SPIs use a standard intake form to collect information from HCWs and provide expert guidance initially using their knowledge and other available resources at the PIC. The primary resource is an online clinical toxicology database called TOXBASE, which was provided to the Nepal PIC free of charge through the National Poisons Information Service Edinburgh.^18^ Other resources include the Nepal PIC protocols and the GoldFranks Toxicologic Emergencies Textbook. SPIs have 2 further layers of support available. First, if additional assistance is required, SPIs use a WhatsApp Group called “National Expert Group,” which comprises local emergency medicine, critical care, clinical pharmacologists, and other relevant experts who can guide SPIs’ queries. Second, as a final line of support, SPIs have access to another WhatsApp Group called “International Expert Group,” which includes medical toxicologists from various countries and international institutions who voluntarily respond to SPIs’ queries on demand. After the initial intake, SPIs continue to follow up the patient’s course in the hospital daily (or sooner as needed) until the patient’s final disposition is made or stability is achieved. Throughout provider interactions, SPIs collect data in a secure REDCap database. The data collection tool includes the patient’s demographic information, history, and agent of poisoning, patient’s clinical metrics, including vital signs, laboratory values, and electrocardiogram. Each SPI follows up on the patient’s clinical metrics. The data collection tool was adapted from an existing data collection tool developed and used by the Emory University Medical Toxicology Section.^19^

Any HCW in Nepal who has a patient with suspected or confirmed case of a toxicological exposure can contact the Nepal PIC, which is staffed by SPIs.

Apart from service delivery, the Nepal PIC engages in education and research as well. The PIC hosts monthly national educational webinars led by international medical toxicologists on various pertinent topics. Recent national topics included the overall approach to toxicological emergencies, paracetamol poisoning, organophosphates, and heavy metal toxicity. Active research projects from the PIC include case reports and building a geographic information system to map the availability of antitoxins and antivenoms in Nepal. Additionally, SPIs hold daily operational meetings with the logistics partner, and the PIC conducts weekly meetings with all relevant partners for quality improvement, case discussions, and logistics.

### Logistics

The Nepal PIC is located within the TUTH Clinical Pharmacology Department, which previously housed the Drug Information Unit. ASK Foundation provided financial and in-kind support for essential equipment for the PIC, including phones, desks, chairs, and computers. Research and salary support for SPIs’ costs is supported by the PI’s grant. The phone number for the Nepal PIC is widely disseminated to HCWs across Nepal through various channels, including social media platforms, monthly webinars, media outlets, and individual phone calls and letters to hospitals across the country. The inauguration event for the PIC was held at TUTH, where stakeholders, including representatives from the World Health Organization, the Nepal Ministry of Health and Population, media representatives, and other national and international stakeholders, were invited and present. The country’s minister of health gave the keynote speech at the event, and the country’s prime minister sent a letter message of support.

### Current Service Update

**FIGURE 3:**
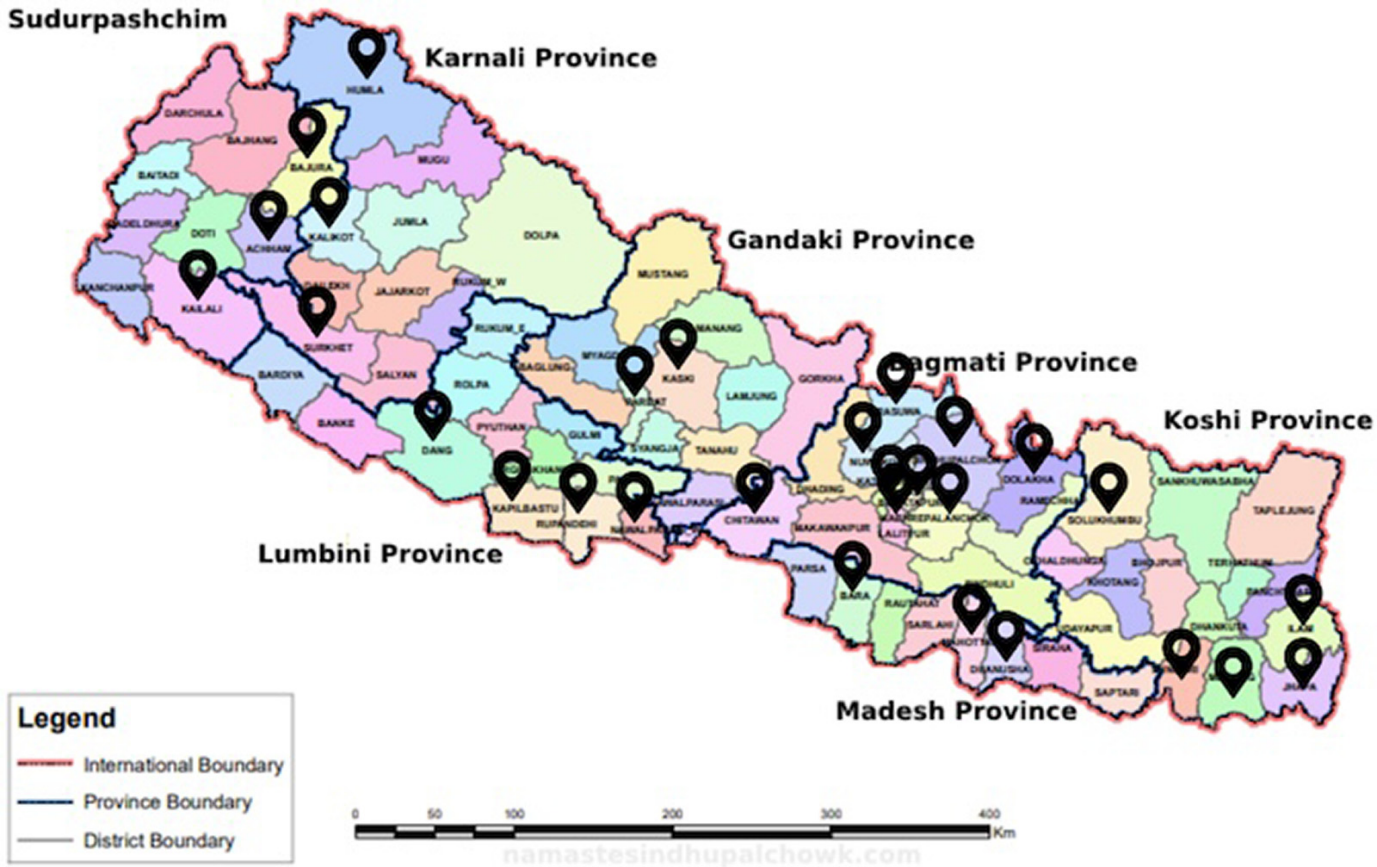
Map of Nepal With Provinces From Which Poison Information Center Has Received Calls^a^ ^a^Pins indicate districts of Nepal from which the Nepal Poison Information Center has received calls from health care workers seeking advice.

**FIGURE 4:**
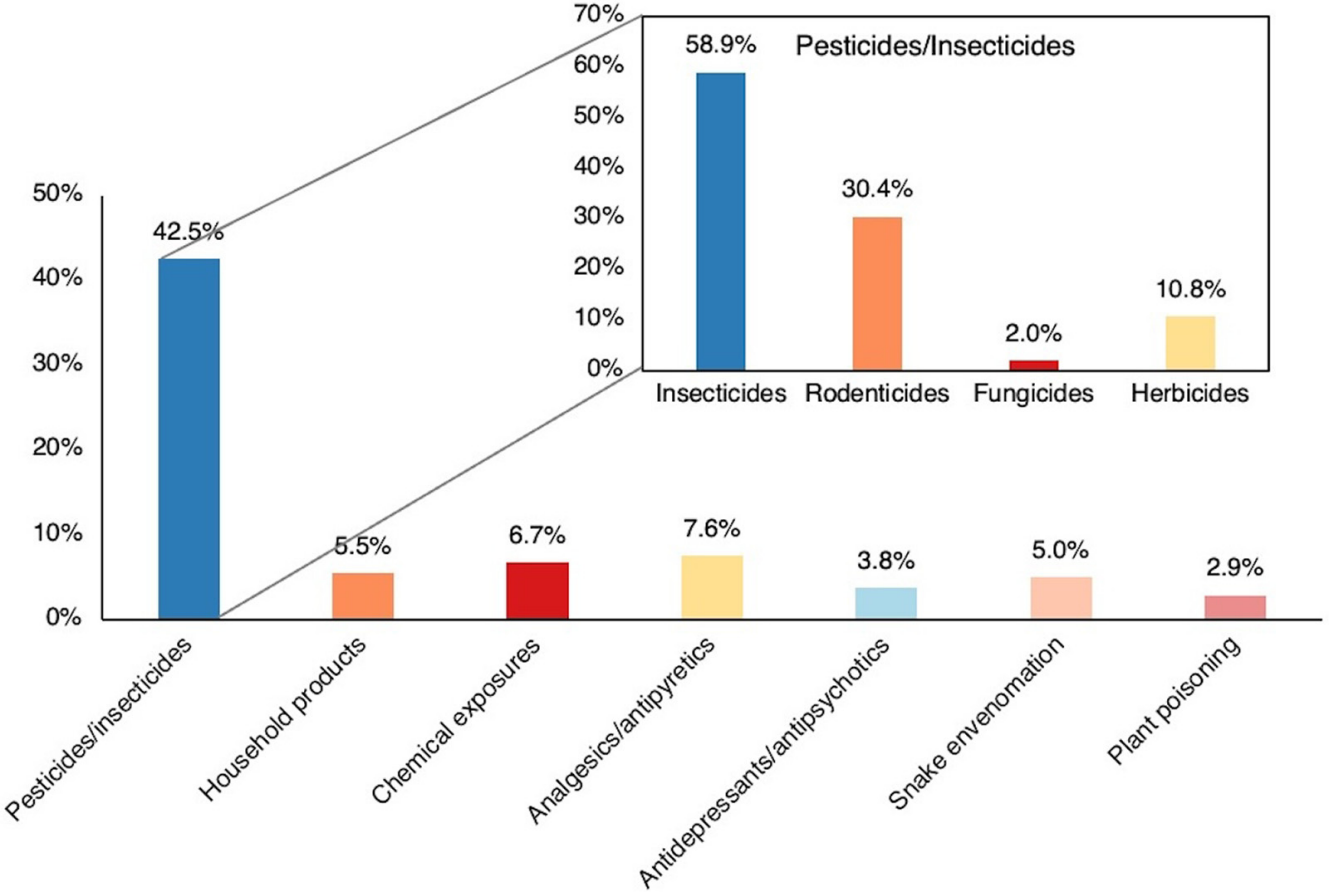
Types of Calls/Cases Received at the Nepal Poison Information Center

The Nepal PIC was inaugurated on December 6, 2023. As of July 17, 2024, the PIC has received calls from all provinces of Nepal (Figure 3). The PIC has provided consultations for nearly 226 patients from across the country, with most calls originating from emergency departments. Of these, 45% of the cases were pesticide exposure, with organophosphates (OP) being the most common. The most common organophosphates were chlorpyrifos (with cypermethrin combination), malathion, dimethoate, and dichlorvos. Other common pesticides/insecticides included zinc phosphide, cypermethrin, and aluminum phosphide. Other calls for consultation involved exposures to antiepileptics, antidepressants, antipsychotics, analgesics/antipyretics, and household products. The PIC received calls for other regional and unique products like “wild mushrooms” and “mad honey”—honey made from bees that extract gryanotoxin from a type of rhododendron flower commonly found in Nepal.^20^ With support from the PIC, 97% of the cases have documented recovery, while 3% resulted in mortality. Mortality was observed in cases involving aluminum phosphide (a rodenticide), paraquat, methanol, and a multidrug overdose. Nearly 26% of all cases have required support from the international expert group. Figure 4 summarizes the cases received.

During the pilot phase, the Nepal PIC has provided consultations for nearly 226 patients from across the country.

### Future Directions and Sustainability

While poison centers generally begin as information centers that provide information to HCWs and the public on various poisonings, they can provide a wide range of other functions.^21^ The Nepal PIC currently provides information to HCWs only and participates in basic research and education endeavors, and there is significant room to expand its functions. In the upcoming months, the Nepal PIC will have an active geographic information system mapping of antivenom availability across the country. After the pilot phase is complete, the next immediate expansion of the PIC will be to take calls from the public. Eventually, the goal is to evolve this into a comprehensive toxicology center with the availability of inpatient toxicology beds, in-person consultations, training programs for clinical toxicology, and continued research. The primary reason for identifying a strong academic partner in Nepal was for its sustainability. TUTH is a government institution and has the advocacy influence within the Ministry of Health and Ministry of Education to seek government-level support for the PIC’s sustainability. Other avenues for short-term sustainability come from the local nonprofit partner that can fundraise for the PIC. The international partners’ involvement allows opportunities for grant funding for other educational and research endeavors through the PIC. Ultimately, the Nepali government must be presented with economic and health benefit data and urged to fund the Nepal PIC. The future direction of Nepal PIC research will include a cost-benefit and econometric analysis. This pilot aims to collect and present data to urge the government’s adaptation of the Nepal PIC.

## DISCUSSION

We present the process of establishing the first institution-based PIC in Nepal. Nepal is home to nearly 30 million people, with a majority rural population and 20.3% living below the poverty line.^22^ Although Nepal’s constitution guarantees the right to free basic health and emergency services, the country’s health system has significant shortfalls in service availability, and the majority of health care expenditure continues to be out of pocket.^23^^,^^24^ The country’s health system is considered one of the weakest in the world. Based on previous evidence from poison centers, this initiative is expected to have not only health benefits but also economic benefits for the health system and the population. The PIC has disseminated its information rapidly across the country through various advertising methodologies. To date, the PIC has served both rural providers in decisions for local monitoring and choosing a local transfer and providers at higher-level facilities in managing complicated toxicology patients with backup support from international toxicology faculty.

In its early stages, the Nepal PIC has shown promise in providing on-time clinical support to health care providers across the country, as well as a potential hub for toxicology education, research, and capacity-building in the country. Due to the nature of its partnership involving numerous international faculty, institutions, and poison centers, international experts are available for a monthly webinar where all health care providers are invited to join from Nepal. These lectures are uploaded to an open-access platform (YouTube). Furthermore, the PIC SPIs and other affiliated individuals have opportunities to join education sessions at various U.S. institutions and poison centers virtually. Building the capacity of Nepali HCWs in medical toxicology is one of the most important tasks the PIC can undertake. After the pilot study, it is vital to continue the PIC service along with education and research as priorities.

There were a few notable challenges in the establishment of the Nepal PIC. Finding the most appropriate collaborator required nearly a year of effort, involving meetings with various partners across Kathmandu. Though there was significant interest from different drug information units to collaborate for the PIC, TUTH provided the faculty support and the academic environment necessary for the PIC’s success, as well as a promise of sustainability. Multiple meetings were held with the lead from the previously existing private entity called the Nepal Drug and Poison Information Center to potentially collaborate with the Nepal PIC, but this effort was not successful. Another major challenge was obtaining a 4-digit toll-free number for the PIC. Due to various bureaucratic hurdles and despite a 6-month effort from the team, the PIC was not able to obtain a 4-digit phone number. One anticipated challenge was the lack of expert support for SPIs when needed. However, volunteer experts have been willing and consistently available to join the group, so the PIC has been able to overcome this challenge for now. This is a potential long-term challenge, and it is vital to build in-country capacity in medical toxicology expertise. There are continued bureaucratic challenges associated with a governmental academic institutional partnership that the Nepal-based partners face.

The Nepal PIC has shown promise in providing on-time clinical support to health care providers across the country, as well as a potential hub for toxicology education, research, and capacity-building in the country.

Six months since its inauguration, the Nepal PIC has supported nearly 250 cases. Despite initial challenges in implementation, this PIC model shows early feasibility in Nepal’s context. There is a desperate need to sustain a PIC in Nepal, and the involvement of the Nepali government is vital. The Nepal PIC leadership team has engaged with the Ministry of Health and Population on various fronts in advocating for further budget for the PIC and for dissemination purposes. There are future opportunities with the ASK Foundation in fundraising and public dissemination. Future efforts of the Nepal PIC will focus on sustainability models and financing advocacy, research, and education, as well as expanding service.

## CONCLUSION

The Nepal PIC was established in response to a mandate from the Nepal government, the high burden of toxicological emergencies in Nepal, and the known economic and health benefits of PICs. Since its inauguration, the Nepal PIC has demonstrated a functional model for providing services and building local capacity in clinical toxicology through multiple national and international partnerships. The future scope of the Nepal PIC includes sustaining its current services and expanding services to the public, expanding toward a comprehensive toxicology treatment center, increasing education and research efforts, and expanding service activities. The field of clinical toxicology is at its infant stages in Nepal, and the Nepal PIC can serve as a focal point in its advancement. Ultimately, the government will need to assume ownership for the long-term sustainability of the Nepal PIC.

## Notes

**Peer Reviewed**

**First Published Online:** September 3, 2024.
